# Localization of Thioredoxin-Interacting Protein in Aging and Alzheimer’s Disease Brains

**DOI:** 10.3390/neurosci3020013

**Published:** 2022-03-31

**Authors:** Haruka Tsubaki, Anarmaa Mendsaikhan, Undral Buyandelger, Ikuo Tooyama, Douglas G. Walker

**Affiliations:** 1 Molecular Neuroscience Research Center, Shiga University of Medical Science, Otsu 520-2192, Japan; ds111857@g.shiga-med.ac.jp (H.T.); anarmaa.mendsaikhan@emory.edu (A.M.); undraa.b12@gmail.com (U.B.); kincan@belle.shiga-med.ac.jp (I.T.); 2 Department of Pharmacology, Emory University School of Medicine, Atlanta, GA 30322, USA; 3 Neurodegenerative Disease Research Center, Arizona State University, Tempe, AZ 85281, USA

**Keywords:** oxidative stress, inflammation, inflammasome, neuropathology, immunohistochemistry, human tissue, amyloid plaque, microglia

## Abstract

Thioredoxin-Interacting Protein (TXNIP) has been shown to have significant pathogenic roles in many human diseases, particularly those associated with diabetes and hyperglycemia. Its main mode of action is to sequester thioredoxins, resulting in enhanced oxidative stress. The aim of this study was to identify if cellular expression of TXNIP in human aged and Alzheimer’s disease (AD) brains correlated with pathological structures. This study employed fixed tissue sections and protein extracts of temporal cortex from AD and aged control brains. Studies employed light and fluorescent immunohistochemical techniques using the monoclonal antibody JY2 to TXNIP to identify cellular structures. Immunoblots were used to quantify relative amounts of TXNIP in brain protein extracts. The major finding was the identification of TXNIP immunoreactivity in selective neuronal populations and structures, particularly in non-AD brains. In AD brains, less neuronal TXNIP but increased numbers of TXNIP-positive plaque-associated microglia were observed. Immunoblot analyses showed no significant increase in levels of TXNIP protein in the AD samples tested. In conclusion, this study identified altered patterns of expression of TXNIP in human brains with progression of AD pathology.

## 1. Introduction

Preventing Alzheimer’s disease (AD) is of the highest significance for the health of the aging population. It is a neurodegenerative disease where affected patients show progressive cognitive decline due to degeneration of critical areas of the brain. Amyloid plaques and neurofibrillary tangles as well as brain atrophy with loss of synapses are the hallmarks of AD pathology [1]. There have been a variety of hypotheses about the causes of AD, with the amyloid cascade hypothesis being the most studied. It is believed that the accumulation of amyloid beta (Aβ) peptide leads to neurotoxicity through oxidative stress, inflammation, or direct toxicity. The initiating events of Aβ accumulation have not been resolved, but oxidative stresses arising from inflammation, mitochondrial dysfunction, and vascular and glucose metabolism dysfunction have been implicated as potential mechanisms [2].

Thioredoxin-Interacting Protein (TXNIP) (previously known as vitamin D3-upregulated protein-1 (VDUP1)) has become recognized as a critical pathological coordinator of oxidative stress pathways [3], glucose metabolism dysfunction [4,5], and inflammation pathways associated with the inflammasome complex [6]. Recent studies have implicated TXNIP in the pathogenesis of human neurodegenerative diseases, including AD and Parkinson’s disease (PD) [7,8,9]. The expression of TXNIP is induced by a number of cellular stress mechanisms, but most widely studied has been hyperglycemia and endoplasmic reticulum stress [10,11]. Increased levels of TXNIP have been associated with enhancement of pathology by increasing oxidative stress and inflammation in a number of diseases [12]. TXNIP can directly enhance oxidative stress by binding to and sequestering antioxidant proteins thioredoxin (TRX)1 and TRX2, thus inhibiting their function [13].

TXNIP interacts with TRXs and also the (NOD)-like receptor protein-3 (NRLP3) inflammasome complex [6]. Activation of the NLRP3 inflammasome complex, after binding of TXNIP, by stimuli that include virus and bacterial infections and Aβ peptide results in enhanced expression of inflammatory cytokines [14]. NLRP3 activation can induce cell death through caspase activation, leading to pyroptosis and increased secretion of interleukin (IL)-1, IL-18, and IL-33 [14]. TXNIP has been studied extensively in relation to diabetes, but few studies have focused on its expression in human AD brains or brains of amyloid plaque-developing AD-model transgenic mice [7,15,16]. Increased levels of TXNIP mRNA or protein have been identified in both human AD brains and AD transgenic mice models [7,15,16]. In addition, it was demonstrated in a mutant tau mouse model of AD that addition of verapamil, a calcium channel blocker, significantly downregulated the levels of TXNIP, which correlated with reduction in the levels of tangle-associated phosphorylated tau [17]. Increased TXNIP levels have been detected in the 3xTg plaque- and tangle-developing AD transgenic mouse model although only in 17-month-old mice [16]. In vitro, Aβ-treatment of human HT22 neurons increased TXNIP expression, while genetic downregulation of TXNIP in this model provided significant protection from neurotoxicity [15]. Previous studies of human AD and control brains showed increased TXNIP mRNA and numbers of TXNIP expressing cells in AD brains [7,9].

Our study aimed to make a detailed characterization of TXNIP cellular expression in human brains affected by progressively increasing amounts of Aβ and neurofibrillary pathology using immunohistochemistry techniques. Our results showed marked localized expression in selective neuronal populations in low- and high-pathology non-demented cases, while in AD cases, increased expression of TXNIP was only observed in microglia-like cells associated with Aβ plaques. Contrary to previous studies, protein level measurements did not show significantly increased TXNIP or decreased TRX total protein levels in AD cases [7,9,18]. Although this study is descriptive in nature, it can contribute to understanding if TXNIP has a role in human AD.

## 2. Materials and Methods 

### 2.1. Human Brain Tissue Samples

Human brain samples used in this study were obtained from the Banner Sun Health Research Institute Brain and Body Donation Program (BBDP), Sun City, AR, USA. The operations of the BBDP as part of the Arizona Study of Aging and Neurodegenerative Diseases (AZSAND) have received continuous approval of Institutional Review Boards (IRB) [19]. Written informed consents for collection and use of brain and other tissues for research purposes were obtained from donors or next of kin. Tissue studies in Japan were approved by Shiga University of Medical Science Ethical Committee (Project Certificate no. 29-114). Demographic details of cases used in this study are summarized in Table 1 (immunohistochemistry samples) and Table 2 (immunoblot samples).

### 2.2. Brain Tissue Preservation and Fixation

All brains were processed at autopsy in a standardized manner [19]. The median postmortem interval for autopsies in the BBDP was 3.8 h. After brain removal, the cerebellum and brain stem were separated from the hemispheres, and then, each was sectioned in a frame into 1 cm thick coronal slabs. The hemispheres were divided, with the left hemisphere being frozen on dry ice for storage at −80 °C and the right hemisphere being fixed for 48 h in buffered formalin solution. After fixation, the coronal pieces were rinsed and transferred to a phosphate-buffered solution of 15% glycerol/15% ethylene glycol as cryoprotectant. Brain regions used for subsequent studies were dissected from frozen or fixed coronal slices by experienced neuroanatomists.

### 2.3. Neuropathological Diagnosis Criteria

All donated brains received full neuropathological diagnosis, including reference to pre-mortem clinical history of each case. Consensus clinical and neuropathological criteria were used to diagnose AD in these cases. To assess severity of AD pathology in each case, tissue sections from 5 brain regions (entorhinal cortex, hippocampus, frontal cortex, temporal cortex, and parietal cortex) were stained with Thioflavin-*S*, Gallyas, or Campbell–Switzer histological stains and assessed semi-quantitatively for the density of neurofibrillary tangles and amyloid plaques, with each brain region being ranked on a scale of 0–3. By combining the measures across these 5 brain regions, assessment of AD pathology was ranked on an ordinal scale of 0–15 for plaques and tangles. The two sets of cases used in this study were subdivided into low-plaque non-demented (LPND, also designated LP) (plaque score < 6), high-plaque non-demented (HPND, also designated HP) (plaque score 6–14), and AD with confirmed dementia (plaque score > 12) [20]. The combined details of age, sex, PMI, apoE4 frequency, plaque, tangle, and Braak staging for two separate series of cases used in this study are listed: series 1 for immunohistochemistry (Table 1) and series 2 for protein analyses (Table 2).

### 2.4. Antibodies

Details of primary antibodies used in this study are described in Table 3 below.

### 2.5. Immunohistochemistry

#### 2.5.1. Peroxidase/Diaminobenzidine Immunohistochemistry

Avidin-biotin-horseradish peroxidase enzyme complex (ABC-Vector Laboratories, Burlingame, CA, USA) histochemistry and nickel ammonium sulfate-enhanced diaminobenzidine (DAB) staining was carried out using temporal cortex sections (25 μm) (MTG, middle temporal gyrus) to identify cellular location of TXNIP in relation to pathological structures. Antibodies used in this study are listed in Table 3. Sections were rinsed three times in phosphate-buffered saline containing 0.3% Triton-X100 (PBSTx) (0.1 M Phosphate buffer, pH 7.4, 0.137 M NaCl, 0.3% Triton-X100 (Nacalai-Tesque, Kyoto, Japan)). Antigen-retrieval pretreatment did not enhance staining of TXNIP monoclonal antibody JY2. Sections were blocked in 1% H_2_O_2_ for 30 min and after that rinsed in PBSTx three times. Sections were incubated in primary antibody overnight on a shaker at room temperature (RT). Sections were rinsed three times in PBSTx and incubated in secondary antibody (biotinylated rabbit anti-goat IgG, biotinylated horse anti-mouse IgG (all from Vector Laboratories, Burlingame, CA, USA)) for 2 h at RT. Sections were rinsed three times in PBSTx and incubated in avidin-biotin complex (ABC) for 1 h. After rinsing in PBSTx and in 50 mM Tris-HCl (pH 7.6), sections were developed in nickel ammonium sulfate-enhanced diaminobenzidine as substrate to produce a purple reaction product (50 mM Tris-HCl, pH 7.6, 1% saturated nickel ammonium sulfate, 40 mM imidazole, 100 μg/mL diaminobenzidine-HCl (Dojindo, Kumamoto, Japan) and 0.0003% hydrogen peroxide). For two-color immunohistochemistry, reacted sections were rinsed in PBSTx, treated with 1% hydrogen peroxide to remove residual peroxidase activity, and then incubated for a second time in primary antibody overnight at room temperature. The detection procedure followed the above-described protocol except the substrate used was diaminobenzidine without nickel ammonium sulfate as substrate to produce a brown reaction product (50 mM Tris-HCl, pH 7.6, 20 mM imidazole, 200 μg/mL diaminobenzidine-HCl and 0.0006% hydrogen peroxide). Reacted sections were mounted on slides, counterstained in most cases with 0.5% neutral red, dehydrated, cleared, and coverslipped using Permount mounting media (ThermoFisher, Waltham, MA, USA).

#### 2.5.2. Laser Confocal Immunofluorescent Staining

Multi-color fluorescent immunohistochemistry was carried out to examine the co-localization of TXNIP with other antigenic markers. Using a free-floating method, sections were incubated in optimal dilutions of primary antibodies overnight at room temperature (RT). Sections were rinsed three times in PBSTx and then incubated in fluorescent-labeled secondary antibodies depending on the primary antibodies used (Alexa Fluor 488-donkey anti-mouse IgG, Alexa Fluor 488-donkey anti-rabbit IgG, Alexa Fluor 568-donkey anti-goat IgG, Alexa Fluor 568-donkey anti-rabbit IgG, Alexa Fluor 647-donkey anti-mouse IgG, Alexa Fluor 555-donkey anti-chicken IgG, Alexa Fluor 647-donkey anti-rabbit IgG) for 2 h at RT. Sections were rinsed three times in PBSTx, slide mounted, and dried at RT, and then, mounted sections were treated with Sudan black (1% solution in 70% ethanol) for 10 min to quench endogenous tissue auto-fluorescence, dipped into 70% ethanol, incubated in DAPI (Thermo Fisher, Waltham, MA, USA) for 2 min in order to reveal nuclei, rinsed in water, and then coverslipped with immuno-mount fluorescent mounting media (Thermo Fisher, Waltham, MA, USA). Images were taken using a Leica SP8 confocal microscope (Leitz, Wetzlar, Germany). All images shown were z-stacks of multiple scans of total thickness of approximately 5 μm.

### 2.6. Protein Extract Preparation

Immunoblotting was carried out to examine the specificity of TXNIP antibodies and their cellular expression patterns and to quantify relative levels of TXNIP in brain samples. Samples were prepared by sonicating in 5 volumes of RIPA buffer (Thermo Fisher Scientific; 20 mM Tris-HCl, pH 7.5, 150 mM, NaCl, 1% NP40, 1% sodium deoxycholate, 0.1% sodium dodecyl sulfate) added with protease and phosphatase inhibitors (Nacalai-Tesque, Kyoto, Japan). Total protein concentration was determined by MicroBCA assay kit with bovine serum albumin as standard.

#### 2.6.1. Si RNA transfected cells for verification of antibody

Cell extracts from retinal pigment epithelial cell line ARPE-19 (RPE) and THP-1 derived macrophages transfected with siRNA for TXNIP or non-specific control were used to demonstrate TXNIP antibody specificity by immunoblot. These samples were used in a recent publication [21]. The exact preparation of TXNIP siRNA transfected extracts is described in detail in this publication [21].

#### 2.6.2. Cell-Type Specificity

Protein extracts from human brain microglia (MG) and human brain vascular endothelial cells (EC) and astrocytes, the hCMEC/D3 endothelial cell line, differentiated iCell neurons (iPSN), and neurons differentiated from the LAN-5 neuroblastoma cell line were analyzed by immunoblot under the same conditions with the different TXNIP antibodies. Preparation of microglia, endothelial cells, and astrocytes from human postmortem brains has been described in detail [22]. The hCMEC/D3 transformed brain endothelial line was obtained from Merck-Millipore (Temecula, CA, USA) and grown as described previously [23,24]. Induced pluripotent cell-derived neurons (iPSN) were obtained from Cellular Dynamics/FujiFilm (Madison, WI, USA) and cultured on poly-L-lysine/laminin for 7 days according to the supplier’s protocol. LAN-5 human neuroblastoma cells were originally obtained from Dr. R.C. Seeger (University of California, Los Angeles, CA, USA) [25] and cultured in RPMI media with 10% fetal bovine serum (FBS). For experiments, LAN-5 cells were differentiated to a neuronal phenotype for 5 days in RPMI+1%FBS in the presence of 10 μM retinoic acid (Sigma-Aldrich, St. Louis, MO, USA) [25].

### 2.7. Western Immunoblotting Analysis

Antibodies used in this study are listed in Table 3. Western blotting analyses were carried out as previously described [26]. Extracted brain samples were dissolved in 4xSDS gel sample buffer (Wako Chemicals, Osaka, Japan), heated at 80 °C for 5 min, and spun at 14,000 rpm for 3 min. Samples were loaded into 4–20% gradient pre-cast gels (Nacalai-Tesque, Kyoto, Japan). Gel electrophoresis was performed at 150 V for 55 min. Proteins were transferred to nitrocellulose by semi-dry electroblotting. Membranes were blocked with 5% milk in Tris-buffered saline with 0.1% Tween 20 (TBST (Takara Bio, Shiga, Japan)) (20 mM Tris-HCl, pH 7.6, 150 mM NaCl, 0.1% Tween 20) for 1 h at RT, and after that incubated in optimal dilution of primary antibody with 2% milk in TBST overnight at RT with shaking. Membranes were rinsed three times in TBST and incubated in secondary antibody (HRP-labeled anti-rabbit or mouse IgG (ThermoFisher)) at 1:10,000 for 2 h at RT. Membranes were exposed to Chemi-Lumi One Super Chemiluminescent substrate (Nacalai-Tesque) and images captured using FUSION SOLO S (Vilber Lourmat Sté, Collégien, France). All membranes were re-incubated in HRP-conjugated antibody to β-actin (Abcam, Cambridge, UK) for normalization purposes.

### 2.8. Statistical Analyses

Statistical analyses were carried out using Graphpad Prism version 7 (Graphpad software, La Jolla, CA, USA). Comparison between disease groups were analyzed by one-way analysis of variance. As no significant differences were revealed by one-way ANOVA of immunoblots, post hoc tests were not carried out. Statistically significant differences were assumed if *p* < 0.05. 

## 3. Results

### 3.1. Characterization and Validation of TXNIP Antibodies Used

**Figure 1:**
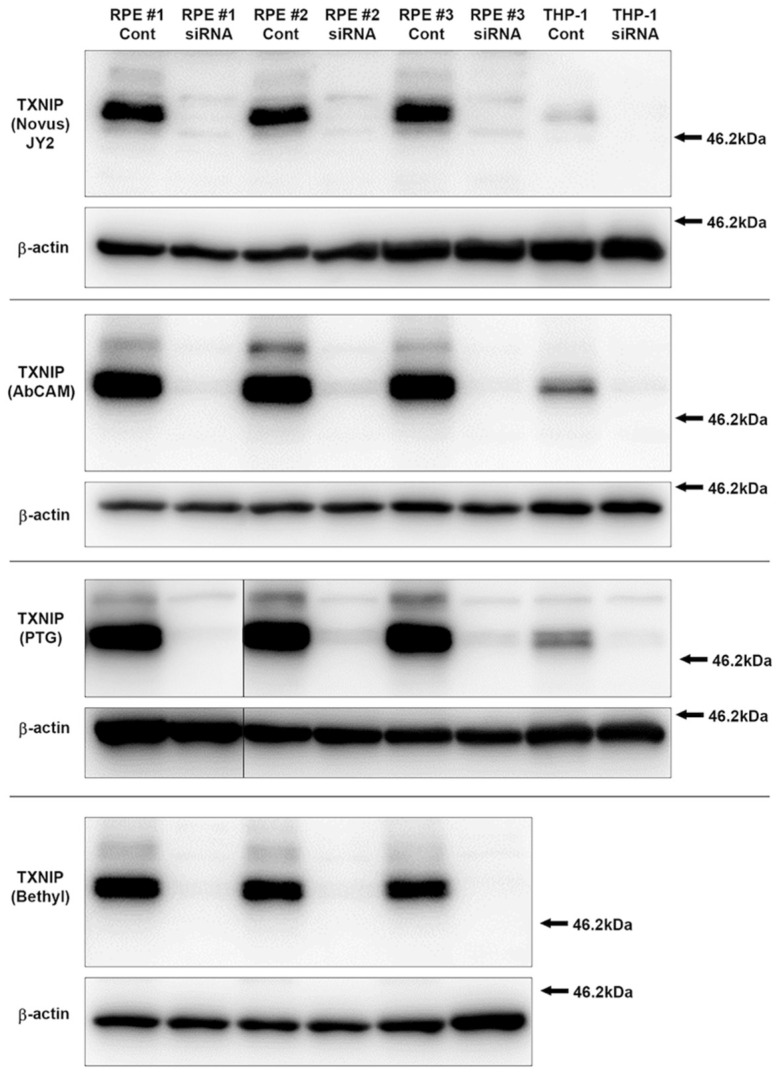
Validation of TXNIP antibodies from different sources using TXNIP specific siRNA knockdown of protein in ARPE-19 retinal pigment epithelial (RPE) and THP-1 macrophages. Composite immunoblots showing matched results using the indicated antibodies. Results demonstrate significant knockdown of bands of 50–55 kDa of the expected molecular weight of TXNIP for all antibodies. Abbreviations: RPE, extracts of ARPE-19 retinal pigment epithelial cells; THP-1, extract of THP-1-derived macrophages; Cont, cell extracts from cells transfected with non-specific control siRNA sequence; siRNA, cell extracts from cells transfected with TXNIP-specific siRNA sequences; kDa, kilodaltons.

The main goal of this study was to characterize in detail the distribution of TXNIP-positive cells in a staged series of human brain samples, including those affected by AD. The choice of antibody to demonstrate TXNIP localization was limited to commercially available antibodies. The characterization of TXNIP/VDUP1 immunolocalization in Drosophila and rat nervous system [2] or in AD brains [7,9] employed the TXNIP monoclonal antibody clone JY2. The origin of this antibody is unclear, but suppliers state it was raised against full-length TXNIP recombinant protein. The exact epitope for this antibody is not known, namely whether it is within the N-terminal domain of TXNIP or in the C-terminal domain that is involved in interactions with TRX. It has not been extensively characterized, but these publications showed it could detect specific bands of 50–55 kDa. Our preliminary studies with this antibody demonstrated a lack of sensitivity for detection of these bands, suggesting it did not have high affinity for detection of denatured TXNIP in immunoblots. For this reason, three other commercial antibodies were also examined: a peptide rabbit antibody produced with a synthetic sequence between amino acids 300–350 (Bethyl Labs, Montgomery, TX, USA); rabbit monoclonal antibody (Abcam) produced against a synthetic sequence between amino acids 50–150; and a rabbit polyclonal prepared against full-length bacterial-expressed TXNIP (Proteintech Group (PTG), Rosemont, IL, US). To verify the specificity of these antibodies, we employed cell extracts from ARPE-19 retinal pigment epithelial cells, which express high levels of TXNIP, and cell extracts from THP-1-derived macrophages that express lower levels. These cells were transfected with control or TXNIP-specific siRNA sequences to demonstrate knockdown of TXNIP expression as described in our previous publication [21]. The results from probing cell extracts transfected with TXNIP siRNA with all four antibodies showed similar large reductions of bands with the expected molecular weights of TXNIP compared to extracts from control siRNA-treated cells (Figure 1), indicating the specificity of each antibody. Minor non-specific bands can also be detected.

**Figure 2:**
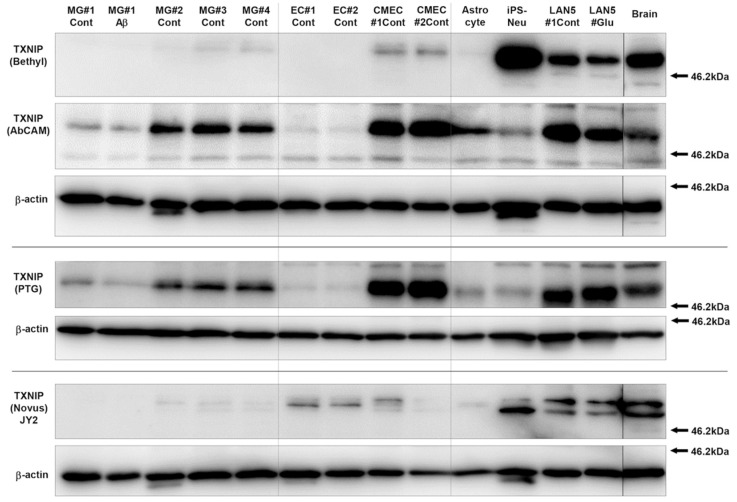
Comparison of TXNIP recognition patterns with antibodies from different sources in different brain-derived cells. Parallel immunoblot membranes with the same amounts of cellular proteins were probed with optimal dilution of four commercial TXNIP antibodies. The results show varying recognition patterns. Equivalent loading between cellular samples is demonstrated with reprobing blots for β-actin. The blot showing results of using AbCAM TXNIP antibody had previously been probed with TXNIP Bethyl antibody Abbreviations: MG, extracts from control (Cont) and Aβ treated microglial extracts from different cases (1,2,3); EC, extracts from primary human brain-derived endothelial cells; MEC, extracts from transformed brain endothelial cell line hCMEC/D3; Astrocyte, extract from brain derived primary astrocytes; iPS-Neu, extracts from Cellular Dynamics iCell human stem cell-derived human neurons; LAN5, extracts from differentiated human neuroblastoma cell line LAN-5 control (Cont) or high-glucose treated (Glu); Brain, extract of human brain samples from AD middle temporal gyrus (MTG).

As the goal of this study was to identify TXNIP expression in brain-derived cells in tissue, immunoblots employing cells extracts from purified microglia, purified endothelial cell, hCMEC/D3 endothelial cell line, human brain-derived astrocytes, and neurons derived from neuronal progenitor cells and differentiated LAN-5 neuroblastoma cells were probed with the four TXNIP antibodies to identify whether there were noticeable differences in specificity for the TXNIP polypeptides expressed by these neural cells. Differences in recognition patterns between the antibodies of detected bands in the different cell types (Figure 2) can be clearly seen. The rabbit monoclonal antibody (Abcam) showed strongest reactivity with the microglial extracts (lanes 1–5), and similar patterns were seen for the PTG TXNIP antibody. The other TXNIP antibodies showed a preference for neuronal and endothelial cells, with lower affinity for microglial-expressed TXNIP. It should be noted that all antibodies recognized the same bands in the brain samples (Brain) as in the neural cell samples. It was observed that Novus TXNIP antibody JY2 could recognize two bands of 50 and 55 kDa in brain and neuronal cell extracts.

### 3.2. Immunoblot Measurements of TXNIP and TRX Levels in LP, HP, and AD MTG Brain Samples

**Figure 3:**
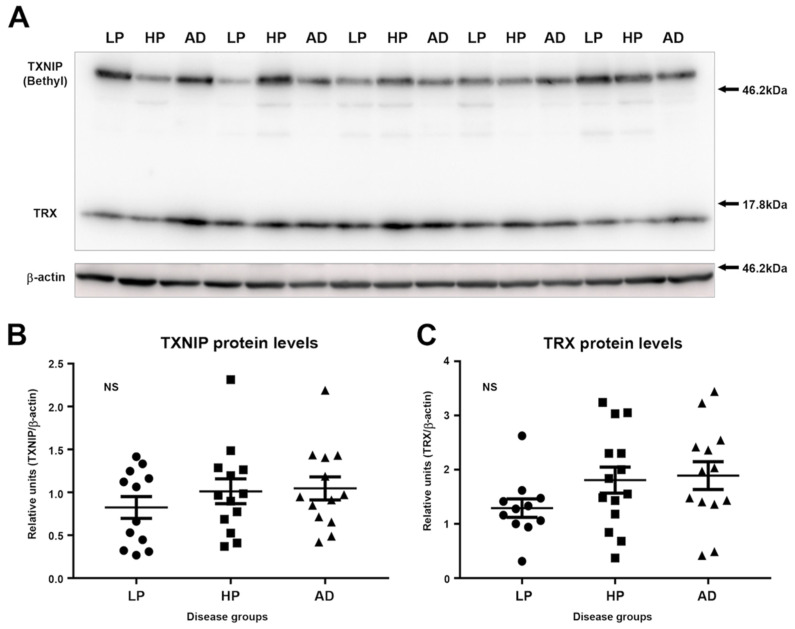
TXNIP and TRX levels in low-plaque (LP), high-plaque (HP), and Alzheimer’s disease (AD) cases. (**A**) Representatives immunoblot of LP, HP, and AD cases showing TXNIP and TRX bands simultaneously on the same membrane. Membranes were probed with rabbit anti-TXNIP (Bethyl, 1:10,000 dilution) and rabbit anti-TRX (Abcam, 1:5000 dilution). Membranes were subsequently reacted with HRP-labeled anti-β-actin antibody (1:15,000) for normalization of loading. (**B**) Relative levels of TXNIP in LP, HP, and AD middle temporal gyrus brain protein extracts. Relative intensities were normalized for β-actin levels. NS, non-significant differences between groups. (**C**) Relative levels of TRX detected in LP, HP, and AD middle temporal gyrus brain protein extracts. Relative intensities were normalized for β-actin levels. NS, non-significant difference between groups.

We carried out immunoblot analyses to measure the relative levels of TXNIP and TRX in LP, HP, and AD MTG brain protein extracts. Due to the large size difference of these proteins, these could be measured simultaneously on the same membrane using an optimized mixture of antibodies. We employed the rabbit polyclonal antibody to TXNIP (Bethyl) or the rabbit monoclonal antibody to TXNIP (Abcam) rather than the Novus monoclonal, as they provided significantly stronger signals. Preliminary experiments had demonstrated that the brain immunoblots had required detection times of 10–15 min to identify measurable bands using the Novus antibody (1:100 dilution), while the other TXNIP antibodies required detection times of approximately 30–60 s under the same conditions. A representative image of TXNIP and TRX polypeptides in selected human samples detected with rabbit polyclonal antibody (Bethyl) are shown in Figure 3A. This was one of the three blots analyzed. Similar results were obtained using the TXNIP rabbit monoclonal antibody (Abcam, data not shown). We did not detect significant differences between the LP, HP, and AD groups for TXNIP or TRX by one-way analysis of variance (Figure 3B or Figure 3C).

### 3.3. Patterns of Expression of TXNIP in Aged and AD Brains

The immunohistochemistry staining presented in this report used the mouse monoclonal antibody JY2 (Novus, Centennial, CO, USA) to identify TXNIP immunoreactive structures. This was the only one of the tested TXNIP antibodies that identified distinct structures in the fixed human brain sections available for this study. In Figure 2, the Western blot patterns for this antibody suggested it did not recognize microglial TXNIP as sensitively as the AbCAM and PTG antibodies. This might have affected the ability to detect immunoreactivity in these cell types. This antibody has been used in previous studies to identify TXNIP expressing cells in human brains and experimental animals [7,9,27].

**Figure 4:**
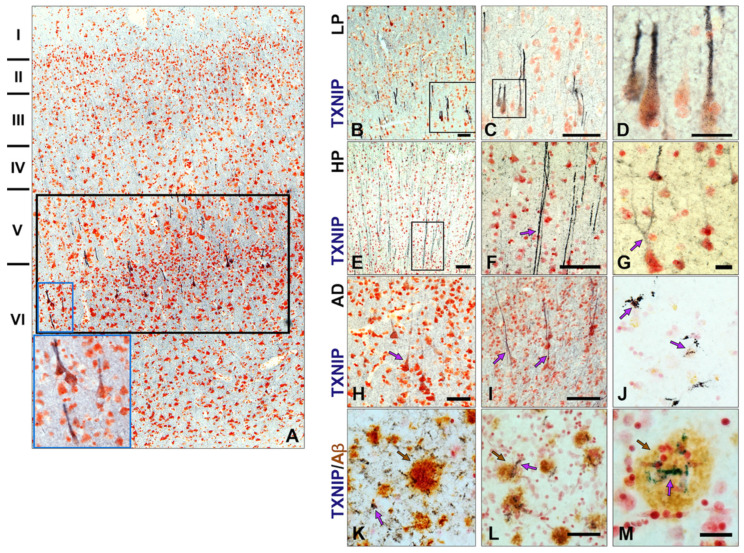
Distribution of TXNIP immunoreactive structures in middle temporal gyrus of LP, HP, and AD cases and in relation to Aβ plaques. (**A**) Low-magnification image of LP case showing selective distribution of TXNIP immunoreactive structures (purple) primarily in layer V and VI (black outline). High magnification inset showing layer VI cell structure (blue outline). (**B**–**D**) Progressively increasing magnification of TXNIP immunoreactive structures (purple) in a low-plaque (LP) case. (**E**–**G**) Different structures showing TXNIP immunoreactivity in high-plaque (HP) cases. (**H**–**M**) TXNIP immunoreactive structures in AD cases. (**H**–**I**). Weaker TXNIP staining of neuronal structures in AD cases. (**J**) Identification of microglia-like TXNIP immunoreactive cells (purple arrow). (**K**–**M**) Identification of microglia-like TXNIP immunoreactive cells (purple arrow) associated with Aβ plaques (brown arrow) in AD cases. TXNIP immunoreactivity is shown in purple color. Aβ immunoreactivity is shown in brown. Abbreviations: LP, low plaque; HP, high plaque; AD, Alzheimer’s disease. Bars represent 50 μm.

Employing tissue from donated brains with short postmortem intervals and short fixation conditions, we observed structures that had not previously been reported to be TXNIP immunopositive. The observed features in sections with progressively increasing pathology are illustrated in Figure 4. We stained sections from all cases listed in Table 1 (low-plaque non demented (LP) *n* = 14, high-plaque non demented (HP) *n* = 11, Alzheimer’s disease (AD) *n* = 13). The rationale for using this series of cases was to characterize differences between aged non-demented brains with sub-pathological levels of plaque and tangle pathology with those from subjects diagnosed with dementia and satisfy the neuropathological diagnosis of AD.

These initial studies employed one- or two-color horseradish peroxidase (HRP) enzyme-based detection immunohistochemistry. Figure 4A illustrates the highly selective location of TXNIP immunoreactive structures, with Figure 4B–M showing different features of cellular morphology. These images represented the typical pattern in the low-plaque (LP), high-plaque (HP) and AD cases examined. It was noticeable that there were selective TXNIP-positive nerve cell processes projecting from cortical layers V–VI (Figure 4A) in the LP case. Figure 4B–D illustrates the location and cellular structures of TXNIP-positive neurons in an LP case. The sections used in Figure 4B–D were derived from a 90-year-old person with no diagnosed cognitive deficit and limited numbers of cortical plaques or tangles. In the sections from HP cases, there were also abundant TXNIP immunoreactive processes (Figure 4E,F) and cells (Figure 4G). By contrast, in the AD brains examined, there were fewer TXNIP immunopositive processes with less-intense staining (Figure 4H,I) and also additional TXNIP-positive cells (Figure 4J).

These cells had the morphology of microglia. Double staining for TXNIP and Aβ (brown) identified additional features of TXNIP expression that were restricted to the AD cases. In Figure 4K–M, TXNIP immunoreactive cells with morphology of microglia can be observed in the vicinity of plaques. In the severe AD case shown, TXNIP immunoreactive microglia were only those that were plaque-associated. These structures were only observed in distinct areas of AD sections and not throughout the section (Figure 4L–M). 

**Figure 5:**
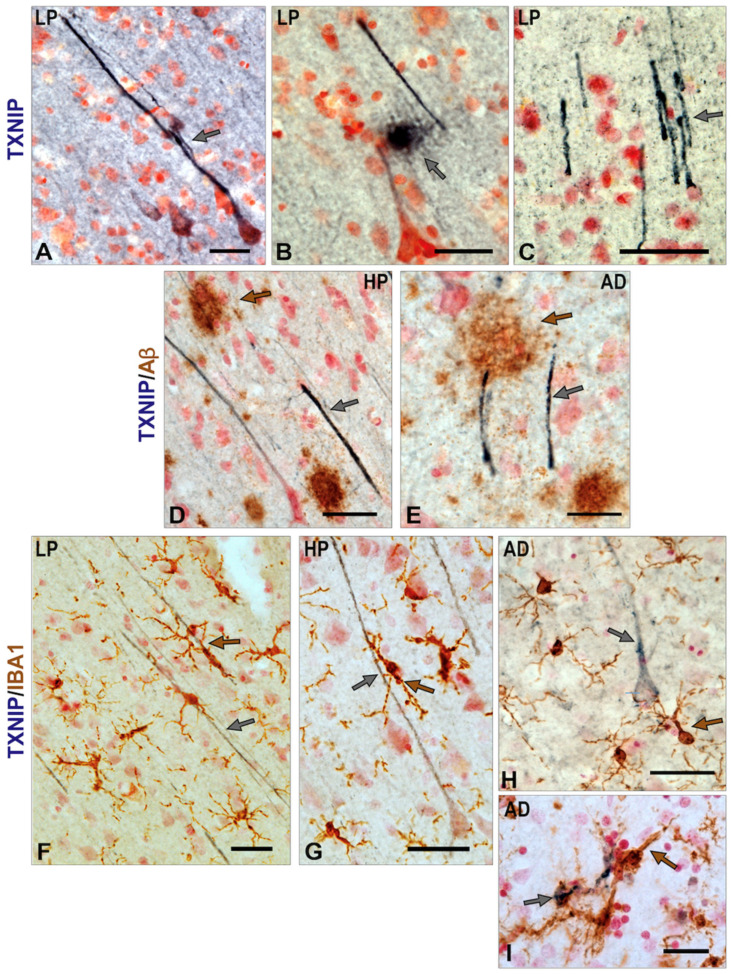
Additional features of TXNIP immunoreactivity in brain samples from middle temporal gyrus. (**A**–**C**) Strongly TXNIP-stained nerve cell (grey arrows) structures present in MTG samples of LP case. Panel B also shows strongly stained extracellular deposits immunoreactive for TXNIP (grey arrow). (**D**,**E**) TXNIP immunoreactive processes (grey arrows) are closely localized in regions with Aβ plaques (brown arrows). (**F**–**I**) Interactions of TXNIP and microglia. (**F**) IBA-1-positive microglia (brown) associated with TXNIP-positive neuronal processes (grey arrow). (**G**) IBA-1-positive microglia (indicated by brown arrow) associated with TXNIP-positive cells (indicated by grey arrow). (**H**) IBA-1-positive microglia (brown) associated with TXNIP-positive neuron processes (grey arrow). TXNIP immunoreactivity (purple). (**I**) Colocalization of TXNIP-positive structures (grey arrow) in IBA-1-positive microglia (brown) in AD case. This feature was rarely observed. Aβ or IBA1 immunoreactivity (brown). Bars represent 50 μm.

Further features of TXNIP immunoreactivity in human brains are presented in Figure 5. Strongly immunoreactive neurons and processes can be observed in certain brain regions, and there was stronger staining in the HP non-demented cases with significant plaque and tangle pathology. Figure 5A–C shows TXNIP staining in a 93-year-old LP case, including the presence of a TXNIP immunoreactive plaque-like structure (Figure 5B). Some TXNIP immunopositive processes were noticeable in areas of brain sections of HP (Figure 5D) and AD cases (Figure 5E) in close association with Aβ immunoreactive plaques. This suggested that increased TXNIP might be a response to localized cellular stress induced by Aβ (Figure 5D–E). There were also noticeable interactions of microglia with TXNIP immunoreactive neuronal processes and cell bodies (Figure 5F–H). It was possible to observe some IBA-1-positive microglia with TXNIP immunoreactivity (Figure 5I), but these were a rare feature (Figure 5I).

### 3.4. Patterns of Expression of TXNIP (Confocal Microscopy)

**Figure 6:**
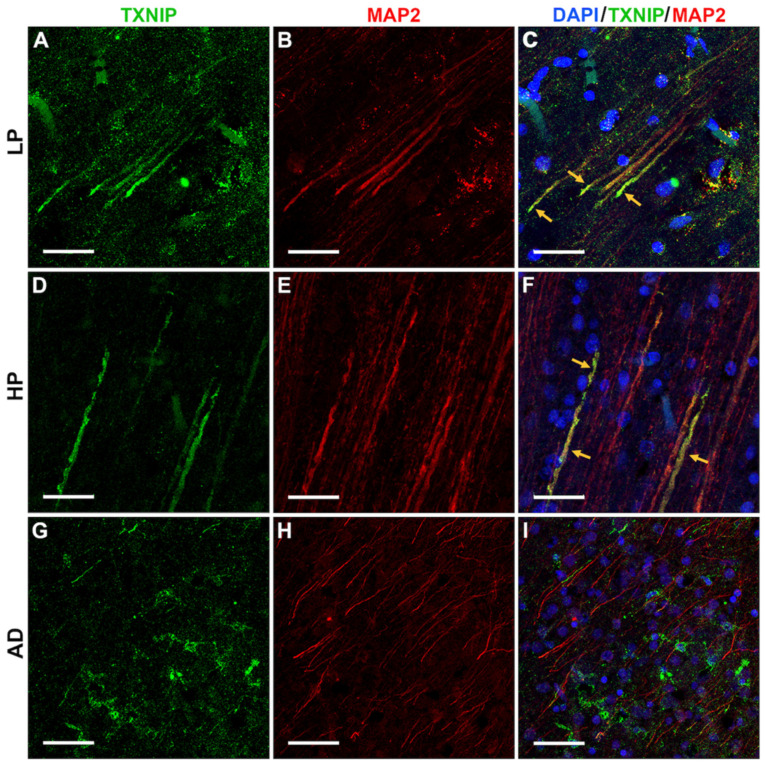
Confocal microscopy showing extensive colocalization of TXNIP with MAP2 immunoreactivity. All sections shown were from middle temporal gyrus. (**A**–**C**) LP case showing (**A**) TXNIP immunoreactivity (green), (**B**) MAP2 immunoreactivity (red), and (**C**) merged image (yellow arrows) with DAPI staining (blue). (**D**–**F**) HP case showing (**D**) TXNIP immunoreactivity (green), (**E**) MAP2 immunoreactivity (red), and (**F**) merged image, indicated by yellow arrows with DAPI staining (blue). (**G**–**I**). AD case showing (**G**) TXNIP immunoreactivity (green), (**H**) MAP2 immunoreactivity (red), and (**I**) merged image showing TXNIP-positive cells with limited colocalization with MAP2 with DAPI staining (blue). Bars represents 30 μm.

**Figure 7:**
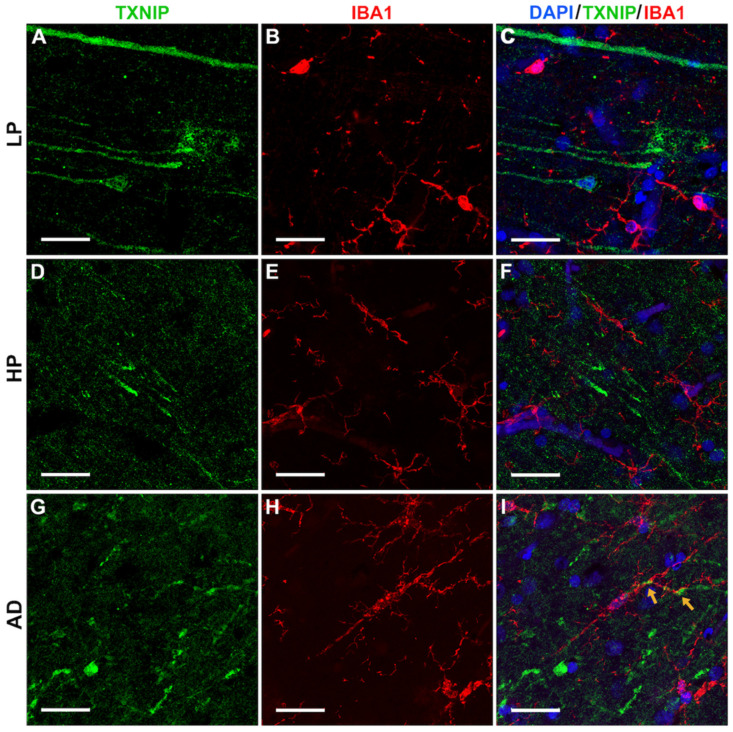
Confocal microscopy showing limited colocalization of TXNIP with microglia marker IBA-1 immunoreactivity. All sections shown were from middle temporal gyrus. (**A**–**C**) LP case showing (**A**) TXNIP immunoreactivity (green), (**B**) IBA-1 immunoreactivity (red), and (**C**) merged image with DAPI staining (blue). (**D**–**F**) HP case showing (**D**) TXNIP immunoreactivity (green), (**E**) IBA-1 immunoreactivity (red), and (**F**) merged image with DAPI staining (blue). (**G**–**I**) AD case showing (**G**) TXNIP immunoreactivity (green), (**H**) IBA-1 immunoreactivity (red), and (**I**) merged image showing TXNIP-positive cells (green) with limited punctate intracellular colocalization (yellow arrow) with IBA-1 (red arrow) with DAPI staining (blue). Bars represents 30 μm.

To confirm the identity of the TXNIP positive structures identified using peroxidase immunohistochemistry staining, which mainly appeared to be in neurons, we carried out fluorescent staining for TXNIP with a range of different markers, including microtubule-associated protein-2 (MAP2), a marker for neuronal dendrites. The results showed that many of the TXNIP-positive structures were co-stained with MAP2, which are indicated by yellow arrows in LP and HP cases (shown in Figure 6A–C (LP) and Figure 6D–F (HP)), but less so in AD cases, where other cells were TXNIP-positive (Figure 6G–I). The AD sections showed noticeably reduced MAP2 staining. We co-stained sections with antibodies to TXNIP and IBA-1 (marker for microglia). However, we did not observe extensive TXNIP co-localization with IBA-1 in most of the images captured (Figure 7) with the exception of AD cases (Figure 7I) (yellow arrows). 

**Figure 8:**
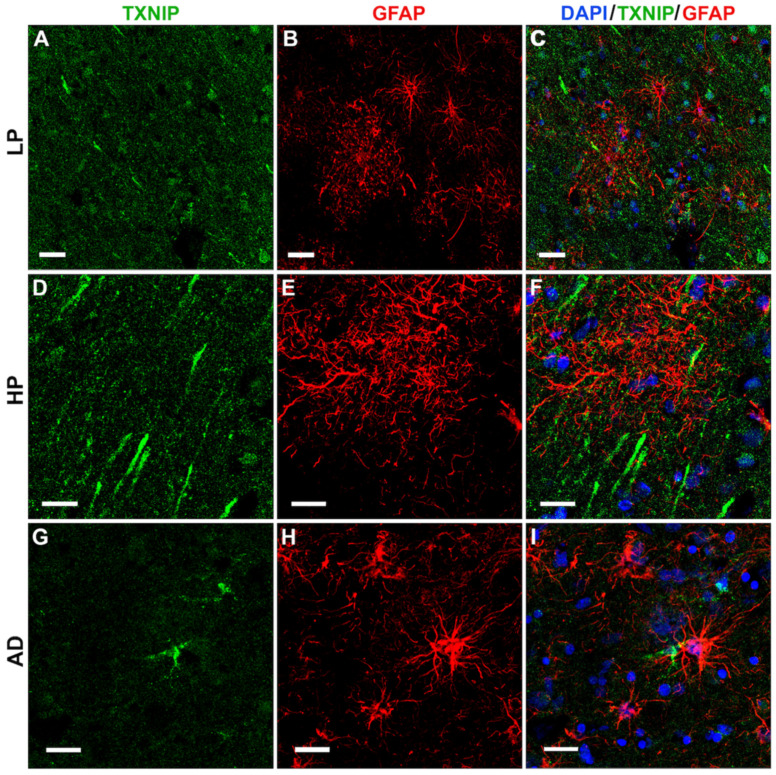
Confocal microscopy showing limited colocalization of TXNIP with astrocyte marker GFAP. All sections shown were from middle temporal gyrus. (**A**–**C**) LP case showing (**A**) TXNIP immunoreactivity (green), (**B**) GFAP immunoreactivity (red), and (**C**) merged image with DAPI staining (blue). (**D**–**F**) HP case showing (**D**) TXNIP immunoreactivity (green), (**E**) GFAP immunoreactivity (red), and (**F**) merged image with DAPI staining (blue). (**G**–**I**) AD case showing (**G**) TXNIP immunoreactivity (green), (**H**) GFAP immunoreactivity (red), and (**I**) merged image with DAPI staining (blue). Bars represents 30 μm.

Immunohistochemical staining of LP, HP, and AD tissue sections was also carried out using antibodies to glial fibrillary acidic protein (GFAP) and TXNIP to verify if colocalization of TXNIP could be observed in astrocytes. Figure 8 demonstrates that TXNIP immunoreactivity did not colocalize with GFAP-positive astrocytes though staining was closely opposed to these different types of astrocytes. This was observed in LP cases (Figure 8A–C), HP cases (Figure 8D–F), and AD cases (Figure 8G–I).

### 3.5. Additional Features of TXNIP Immunoreactive Structures (Confocal Microscopy)

**Figure 9:**
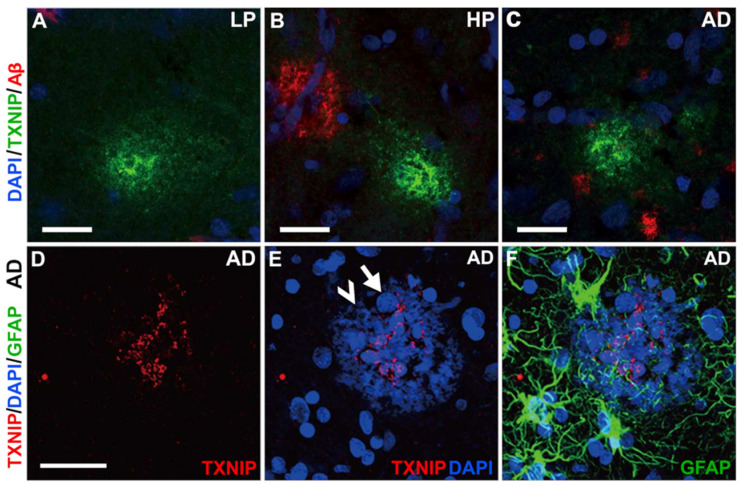
TXNIP immunoreactive plaque structures. (**A**–**C**). TXNIP immunoreactive plaque-like structures (green) present in LP (**A**), HP (**B**), and AD cases (**C**) do not colocalize with Aβ (red). Bars represent 20 μm. (**D**–**F**). Some mature plaques have TXNIP immunoreactivity (red) (**D**). Plaque revealed by accumulations of DAPI staining of nuclei (white arrow) but also amyloid-like material (white arrowhead) structures (**E**). Plaque-associated GFAP-positive astrocytes (green) accumulated around plaque-like structures and did not show TXNIP immunoreactivity (**F**). Bars represent 30 μm.

This section will consider additional features of TXNIP immunoreactive structures in relation to pathological features. Figure 9 illustrates types of TXNIP immunoreactive plaque-like structures.

Figure 9A–C shows that plaque-like accumulations of TXNIP were present in sections from all disease groups. These structures referred to in Figure 5B are distinct from Aβ immunoreactive plaques (red in panels B and C). In Figure 9D,E of an AD case, the TXNIP-positive structure (red) colocalized with a plaque-like structure. This was revealed using DAPI staining that not only recognized accumulations of nuclei as expected (white arrows) but also appeared to bind to highly aggregated amyloid plaque (white arrowheads) [28]. The TXNIP-positive structures did not colocalize with GFAP-positive astrocytes around the plaques.

**Figure 10:**
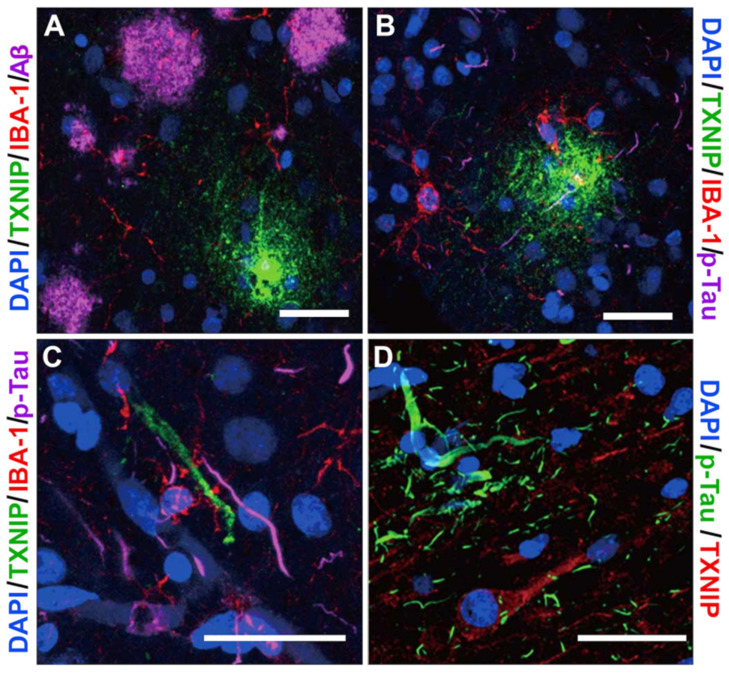
Interactions of TXNIP with pathological features in middle temporal gyrus (**A**–**C**). Triple staining demonstrating TXNIP (green), IBA-1 (red), and Aβ (A, purple) or p-Tau (B–C, purple). (**D**) Absence of colocalization of p-Tau (green) and TXNIP (red). Bars represent 30 μm.

Further characterizations of TXNIP immunoreactive structures were made using triple immunohistochemistry staining. Figure 10A shows no overlap between Aβ plaque (purple) and TXNIP plaque-like deposits (green). These results confirm that these TXNIP plaque-like structures do not colocalize with Aβ, but in Figure 10B, microglia (red) can be seen interacting with these TXNIP structures. In contrast, Figure 10C,D demonstrate TXNIP did not colocalize with p-Tau-positive structures. 

## 4. Discussion

The aims of this study were to identify the cellular expression and distribution of TXNIP in human temporal cortex in tissue samples with different degrees of plaque and tangle pathology. We had hypothesized that TXNIP would be mainly localized to microglia but observed immunolocalization in neuronal dendrites in cases defined as non-demented but with low or high amounts of plaque and tangle pathology. The neuronal immunoreactivity for TXNIP was reduced in the AD cases, but these did show limited numbers of TXNIP-expressing, microglia-like cells around plaques. In addition, we measured the levels of TXNIP protein in these samples by Western blot analyses to determine if this protein was increased with disease but found no significant difference between the disease groups.

A number of studies have shown how increased expression of TXNIP makes significant contribution to disease processes, particularly those related to diabetes, but few have related TXNIP to human brain diseases (reviews: [29,30]). The major property of TXNIP is to bind and sequester the antioxidant thioredoxin (TRX), which results in increase in damaging oxidative stress with pathological consequences. A second function of TXNIP, which can be related to or independent from its TRX binding, is to activate the NLRP3 inflammasome complex, resulting in enhanced inflammation.

Previous immunohistochemistry studies of normal animal brains have shown that TXNIP was expressed by neurons, astrocytes, and microglia, with overall expression levels in the brain appearing low compared to other tissues [27]. Under pathological conditions, an animal model of cerebral ventral sinus thrombosis demonstrated significant induction of TXNIP in neurons and in some astrocytes and microglia but restricted to sites of acute neuronal cell death. Of note from this study was that the increase in TXNIP was time-limited, peaking at 3 days after lesioning, and then resolving to baseline levels [31]. These data imply that increased TXNIP could be restricted to sites of active pathology. Two other studies that employed the animal model of subarachnoid hemorrhage showed that increased TXNIP expression correlated with enhanced tissue damage, while blocking TXNIP expression with resveratrol or specific siRNAs reduced tissue damage [11,32].

Two recent studies examined the expression of TXNIP in human AD brains. These studies employed different brain regions (frontal cortex and hippocampus) from the brain region used to produce results in this report (temporal cortex). These other studies showed increased expression of TXNIP mRNA in AD brains and increased numbers of TXNIP immunoreactive cells but no increase in protein levels [7,9]. Our results demonstrated that TXNIP protein levels in MTG were not markedly different between AD and aging non-AD patients but did not measure mRNA levels. In this study, compared to the above-mentioned papers, we categorize the non-AD cases into low-plaque and high-plaque features. The study reported here focused on fully characterizing cellular expression of TXNIP in relation to pathological structures in human brains. Using the same JY2 TXNIP antibody as previously reported, we identified differential expression of TXNIP in neuronal dendrites and some neuronal cell bodies, particularly in cortical layers V–VI of the low- and high-pathology, non-demented cases. TXNIP immunoreactive structures did not colocalize with p-Tau immunoreactive tangles but in the high-plaque cases appeared to be in areas where plaques develop. These neuronal structures colocalized with MAP2, a marker for neuronal dendrites. The expression of neuronal TXNIP appeared to decrease in AD cases, while TXNIP expression in subsets of microglia, particularly those associated with plaques, could be seen in AD cases. It was noticeable that only a limited number of microglia-like cells showed TXNIP immunoreactivity. It has previously been observed using an AD plaque-developing mouse model (APP/PS1) that TXNIP protein levels were increased in frontal cortex and hippocampus of 9- and 12-month-old mice compared to wild-type non-transgenic mice, while TRX levels were unchanged [15]. This study did not examine the cellular distribution of TXNIP in these animals. However, using a neuronal cell line, Aβ treatment of cells resulted in increased levels of TXNIP, confirming Aβ as a TXNIP-inducing stress factor [15]. The expectation of significant microglial expression of TXNIP came from recent single-cell RNA profiling studies of microglia that defined TXNIP as a microglial activation marker [33,34].

One limitation of this study, common with other immunohistochemistry studies, might be the specificity of the antibodies used to demonstrate cellular localization. Using siRNA, we demonstrated that four different commercial TXNIP antibodies identified the same TXNIP polypeptides of 50–55 kDa, whose detection was inhibited with TXNIP siRNA transfection. The molecular weight of TXNIP can vary depending on degree of post-translational modifications [35]. This demonstrated specificity of all antibodies, but only the mouse monoclonal JY2 antibody showed the staining patterns demonstrated in the figures. The JY2 antibody has been used in previous reports to demonstrate cellular localization of TXNIP [7,9,27]. This antibody, however, was significantly less sensitive in detecting TXNIP by Western blot in brain samples. We did not identify significantly increased levels of TXNIP in AD brain samples. This result was similar to the other recent publications. These authors detected significantly increased TXNIP mRNA in AD cases but non-significant increases in proteins levels [7,9]. It can be seen in Figure 3 that there was considerable variability in TXNIP levels between samples in all disease groups.

The validity of the immunostaining is seen by its selective patterns of expression brain. Non-specific staining would not be expected to be so localized or show the changing patterns between disease groups. Ideally, pre-absorption of antibody with purified protein, resulting in abolishment of staining, is needed; however, purified TXNIP was not available for this study. Due to the different properties of the N-terminal domain compared to C-terminal domain of TXNIP in terms of binding to TRX [36], it might be expected that differences in recognition of the native proteins in fixed tissue sections is more selective than in immunoblots, where the secondary structure of proteins is not a feature due to sample denaturation.

Overall, there can be different interpretations for the altered staining patterns of TXNIP, but it should be considered that increased TXNIP staining in neurons in human brains might be a response to increased cellular stress prior to the development of AD pathological hallmarks. Increased expression of TXNIP in microglia at the later stage of the disease rather than at stages before significant AD pathology has developed suggest that TXNIP-mediated activation of inflammasome-induced inflammation might be occurring at late stages of the disease. Increased NLRP3 expression was detected in AD cases in other studies, where there were no staging of the control cases into low- and high-pathology, non-demented classification [7,9,14]. Due to the significant role of TXNIP in diabetes-related pathologies and on the possible interactions of diabetes and/or insulin resistance in AD pathologies [37,38,39], further studies using brain tissues from cases with and without history of diabetes or insulin resistance would be informative. It was thus a reasonable hypothesis to believe that TXNIP could be involved in AD. There are now significant data that insulin dysregulation due to obesity, aging, or metabolic syndrome exacerbates cognitive decline, possibly due to interfering with Aβ degradative processes. However, the direct enhancement of diabetes on neuropathology has been better demonstrated in AD mice models [40,41,42] than in human AD brains [43,44,45], where findings suggest that diabetes has a significant risk factor for cerebrovascular disease [46]. One significant feature was the identification of apoE4 genotype as a significant risk factor in conjunction with diabetes in enhancing AD-type neuropathology [37,47].

In summary, the findings in this study suggest that TXNIP expression in neurons could identify vulnerable neurons where increased oxidative stress is present. The decline in neuronal staining in AD cases coincides with loss of MAP2 immunoreactivity due to neuronal damage. The results also indicate that TXNIP could not be considered a marker of activated microglia, as numbers of microglia showing TXNIP staining were limited. 

## Figures and Tables

**Table 1:** Demographic details of human brain middle temporal gyrus (MTG) cases employed in immunohistochemistry studies. Series 1 (Abbreviations: LP, low-plaque non-demented; HP, high-plaque non-demented; AD, Alzheimer’s disease; M:F, male:female numbers; SD, standard deviation; PMI, postmortem interval; APOE4, percentage and numbers of cases with APOE4 genotype; SEM, standard error of mean).

	Gender (M:F)	Mean Age ± SD/y	PMI/h	APOE4	Plaque Score ± SEM	Tangle Score ± SEM	Braak Score
LP (*n* = 14)	8/6	85.7 ± 8.1	3.2 ± 1.0	3.8% (1/26)	2.1 ± 0.6	4.8 ± 0.7	I–IV
HP (*n* = 11)	4/7	83.6 ± 6.9	3.0 ± 0.7	20% (4/20)	10.6 ± 0.8	4.5 ± 0.7	III–IV
AD (*n* = 13)	9/4	80.2 ± 6.2	3.5 ± 0.8	38.5% (10/26)	14.3 ± 0.2	13.9 ± 0.5	V–VI

**Table 2:** Demographic information of cases used for immunoblot analysis. Series 2 (Abbreviations: LP, low-plaque non-demented; HP, high-plaque non-demented; AD, Alzheimer’s disease; M:F, male:female numbers; SD, standard deviation; PMI, postmortem interval; APOE4, percentage and numbers of cases with APOE4 genotype; SEM, standard error of mean).

	Gender (M:F)	Mean Age ± SD/y	PMI/h	APOE4	Plaque Score ± SEM	Tangle Score ± SEM	Braak Score
LP (*n* = 12)	7/5	86.8 ± 7.2	2.6 ± 0.6	8.3% (2/24)	1.7 ± 0.62	4.1 ± 0.6	I–IV
HP (*n* = 14)	9/5	83.8 ± 5.5	3.1 ± 0.7	21.4% (6/28)	9.0 ± 0.5	3.5 ± 0.5	I–IV
AD (*n* = 13)	5/8	78.0 ± 9.4	3.0 ± 0.9	38.5% (10/26)	14.3 ± 0.2	13.2 ± 0.8	IV–VI

**Table 3:** The primary antibodies used in this study.

Antigen	Antibody	Supplier	Host Species	Catalog No.	Application	Dilution
TXNIP	TXNIP (JY2)	Novus	Mouse/Mono	NBP1-54578	IHC/WB	1:200–500
TXNIP	TXNIP	Bethyl	Rabbit/Poly	A303-229A	WB	1:10,000
TXNIP	TXNIP	Abcam	Rabbit/Mono	ab188865	IHC/WB	1:2000
TXNIP	TXNIP	Proteintech	Rabbit/Poly	18243-1-AP	WB	1:2000
TRX	TRX	Abcam	Rabbit/Mono	ab133524	IHC/WB	1:1000–5000
β-actin	HRP-β-actin	Abcam	Mouse/Mono	ab49900	WB	1:30,000
Aβ1-42	Amyloid β (mOC64)	Abcam	Rabbit/Mono	ab201060	IHC	1:1000
P-tau	P-tau (S202)	Abcam	Rabbit/Mono	ab108387	IHC/WB	1:3000–5000
P-tau	P-tau (S396)	Abcam	Rabbit/Mono	ab109390	IHC/WB	1:3000–5000
MAP2	MAP2	Abcam	Chicken/Poly	ab92434	IHC	1:1000
GFAP	GFAP	Dako	Rabbit/Poly	Z0334	IHC	1:2000
IBA1	IBA1	Abcam	Goat/Poly	ab5076	IHC	1:1000
